# Short Communication: Is Ethanol-Based Hand Sanitizer Involved in Acute Pancreatitis after Excessive Disinfection?—An Evaluation with the Use of PBPK Model

**DOI:** 10.1155/2012/959070

**Published:** 2012-04-17

**Authors:** Céline Huynh-Delerme, Catherine Artigou, Laurent Bodin, Robert Tardif, Ginette Charest-Tardif, Cécile Verdier, Nessryne Sater, Mostafa Ould-Elhkim, Catherine Desmares

**Affiliations:** ^1^Department of Cosmetic, Biocide, and Tatoo Products, French Health Products Safety Agency (Afssaps), 143-147 Boulevard Anatole France, 93285 Saint-Denis Cedex, France; ^2^Department of Risk Assessment, French Agency for Food, Environmental and Occupational Health Safety (Anses), 24-31 Avenue du Général Leclerc, 94701 Maisons-Alfort Cedex, France; ^3^Département de Santé Environnementale et Santé au Travail, Faculté de Médecine, Université de Montréal, Montréal, QC, Canada 3J7 H3C

## Abstract

An occupational physician reported to the French Health Products Safety Agency (Afssaps) a case of adverse effect of acute pancreatitis (AP) in a teaching nurse, after multiple demonstrations with ethanol-based hand sanitizers (EBHSs) used in a classroom with defective mechanical ventilation. It was suggested by the occupational physician that the exposure to ethanol may have produced a significant blood ethanol concentration and subsequently the AP. In order to verify if the confinement situation due to defective mechanical ventilation could increase the systemic exposure to ethanol *via* inhalation route, a physiologically based pharmacokinetic (PBPK) modeling was used to predict ethanol blood levels. Under the worst case scenario, the simulation by PBPK modeling showed that the maximum blood ethanol concentration which can be predicted of 5.9 mg/l is of the same order of magnitude to endogenous ethanol concentration (mean = 1.1 mg/L; median = 0.4 mg/L; range = 0–35 mg/L) in nondrinker humans (Al-Awadhi et al., 2004). The present study does not support the likelihood that EBHS leads to an increase in systemic ethanol concentration high enough to provoke an acute pancreatitis.

## 1. Introduction

Ethanol in hand sanitizing gel is widely used not only in health care settings but also in other areas that involve hand hygiene. Ethanol is considered an effective substance against a large spectrum of microorganisms which can linger on the skin. Health care systems and infection control organizations have begun advocating the routine use of hand sanitizing gel, as health care professionals may apply alcohol-based hand sanitizers more than 50 times a day, if using these products prior to and just following each patient.

However, as alcohol drinking is associated with an increased risk of a number of cancers, birth defects, or other health disease disorders like pancreatitis, there is no common consensus on the safety of ethanol-based hand sanitizers (EBHSs) in the literature. In its recent opinion, the French Health Products Safety Agency (Afssaps) considered that the use of ethanol as hand disinfectant is safe, taking into consideration the low dermal absorption even after excessive disinfection [1]. Although this absence of risk is established, the Afssaps recommended to consumers to privilege the washing hands with soap and water due to its sufficient microbiological efficacy. Ethanol hand sanitizers should rather be used when soap and water hand washing is not available [2].

In this context, an occupational physician reported to the French Health Products Safety Agency a case of acute pancreatitis (AP) in a 46-year-old teaching nurse. She has been working in a nursing school for seven years. This adverse effect appeared after demonstrations using ethanol-based hand sanitizers for two successive days in a classroom under conditions of defective mechanical ventilation. Knowing the relationship between excessive alcohol consumption and risk factor associated with either acute or chronic pancreatitis, the occupational physician suggested that the exposure to ethanol by hand skin and also mainly *via* inhalation route may have led to a significant systemic ethanol concentration increase and consequently to the AP symptom.

The reconstitution of the events showed the following.

- A two successive-day demonstration (TSDD) with EBHS was carried out in mid-September (2009) without any complaint.
- However, three days after this TSDD, the trainer complained of headaches after having stayed 30 minutes in the office opened on the classroom, in which the demonstration has been carried out.
- Six days after the TSDD, the trainer, seven students, and the manager of local “committee for health, safety and working conditions” suffered from malaise and headache in the same office. The intervention of the firemen excluded possible carbon monoxide intoxication but they noted a strong smell of alcohol. The technical agent who was called in highlighted the ventilation defect.
- Seven days after the TSDD, the trainer had headaches and dizziness again after having stayed two hours in the same premises without any ethanol handling.
- She did not work the ten following days.
- Seventeen days after the TSDD, while at home, she felt severe epigastric pain radiating to the low dorsal after having drunk a glass of red wine.
- Eighteen days after the TSDD, based on the clinical symptoms observed the day before and the biochemical analysis (lipase at 1174 IU/L (range 23–300 IU/L) and an amylase at 142 IU/L (range 25–125 IU/L)), it was suggested an acute pancreatitis diagnosis.
- Thirty days after the TSDD, the biochemical analysis performed again was normal (lipase 187 IU/L and amylase 50 IU/L). Transaminase, aspartate aminotransferase, and alanine aminotransferase were also normal. In the end, the abdominal scan performed was normal.

On the other hand, the medical history shows that the teaching nurse presented a hyperthyroidism treated in early 2009 by NeoMercazole and Levothyrox for 3 months. At that time, lipase and amylase were normal. This treatment was stopped at least one month before the onset of the AP. She has been treated for hypertension with a chlorothiazidic diuretic, Esidrex, for 3 or 4 years. The diuretic was stopped about two months after the first event.

The patient did not take oral contraceptives and she drank alcohol without abuse.

This lead to the question as whether or not EBHS use could have resulted in significant blood ethanol concentration increase. This study was carried out firstly, to predict, by theoretical approach, ethanol concentration in the classroom air following EBHS use under defective ventilation; secondly, to estimate, using a physiologically based pharmacokinetic (PBPK) model, the blood ethanol concentration likely to be reached after ethanol inhalation in the classroom air.

## 2. Materials and Methods

### 2.1. Exposure Conditions

**EBHS Used**

The hand disinfectant contained ethanol at 700 mg/g (755 mL/L) in the presence of thickening, moisturizing and emollient agents, and water. It contained neither perfumes nor dyes. The potential implication of each product ingredient in the manifestation of pancreatitis was also checked.

**Exposure**

Training for auxiliary nurses was conducted for two successive days in the same classroom, 28 students and two trainers were in the classroom. The amount of product used for each friction was 3 mL; with a daily number of frictions per day equal to 3 per person. Thus, the total was 180 frictions (540 mL EBHS, i.e., 378 mL of ethanol) for two days.

**Class Volume**

The classroom volume alone was 116 m^3^. It is opened to another handling room and office; thus, the total volume was 310 m^3^. However, in order to simulate worst case conditions, the total exposure to EBHS of 540 mL for two days was considered only in 116 m^3^.

### 2.2. Exposure Assessment

The air ethanol concentration was estimated using an American Industrial Hygiene Association (AIHA) software [4]. Assessments of exposure to indoor air pollutants usually employ spatially well-mixed models which assume homogeneous concentrations throughout a building or room. The theoretical approach used with AIHA software is based on the description of the spray and the substance behavior but also on the modeling of the ethanol concentrations occurring in homogenous, mixed rooms.

As mechanical ventilation was defective, 0.08 m^3^/min considered as the worst scenario was retained.

Considering the number of frictions, the following scenario was considered: total rubbing hands equal to 180 frictions (3 times/30 persons/2 days) with EBHS, over a period of 48 hours interrupted by a night.

The ethanol atmospheric emission was calculated as follows: 30 rubbing hands (RH) at time 0 h, 30 RH at 3 h, and 30 RH at 6 h (for the first day), and 30 RH at 24 h, 30 RH at 27 h, and 30 RH at 30 h (for the second day), with 3 mL EBHS at 70% ethanol and a density mass of 0.8.

### 2.3. Blood Ethanol Calculation

Blood ethanol concentrations were predicted using a physiologically based pharmacokinetic (PBPK) model, the ACSLX software (Version 3.0.1.6; AEgis Technologies Group, Inc.), which allows simulation of inhalation exposure to various air ethanol concentrations and prediction of its toxicokinetic behaviour [5].

**Figure 1:**
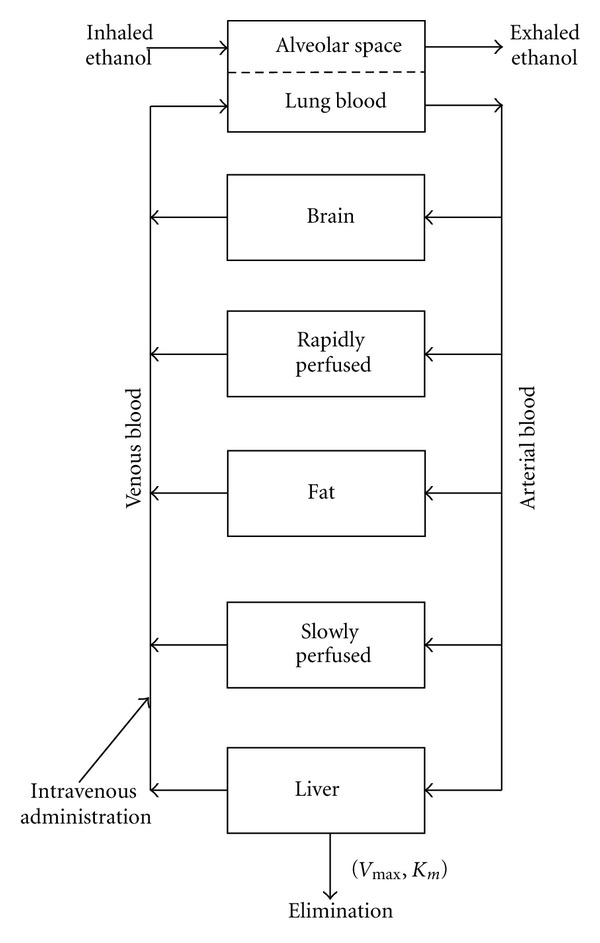
Schematic representation of the ethanol physiologically based pharmacokinetic model (PBPK) proposed by Pastino et al. [3].

The blood flow limited PBPK model for ethanol was previously developed for human by Schlouch and Tardif [6] and for rodent by Pastino et al. [3]. Compartments for the present model include liver, brain, fat, rapidly perfused tissue, slowly perfused tissue and blood. A schematic diagram of the PBPK model for ethanol inhalation is represented in Figure 1. Mass-balance equations were written describing the rate of change in ethanol concentration for each compartment. 

The blood flows and tissue volumes for each compartment (Table 1) were obtained from the report prepared by the United States Environmental Protection Agency (US EPA) on “Physiological Parameter Values for PBPK Models” [7]. The ethanol partition coefficients for rats were determined by Pastino et al. [3].

The fractional uptake in the airways is reported to be mostly between 55 and 62% [3].

## 3. Results

### 3.1. Air Ethanol Concentrations Estimation

**Figure 2:**
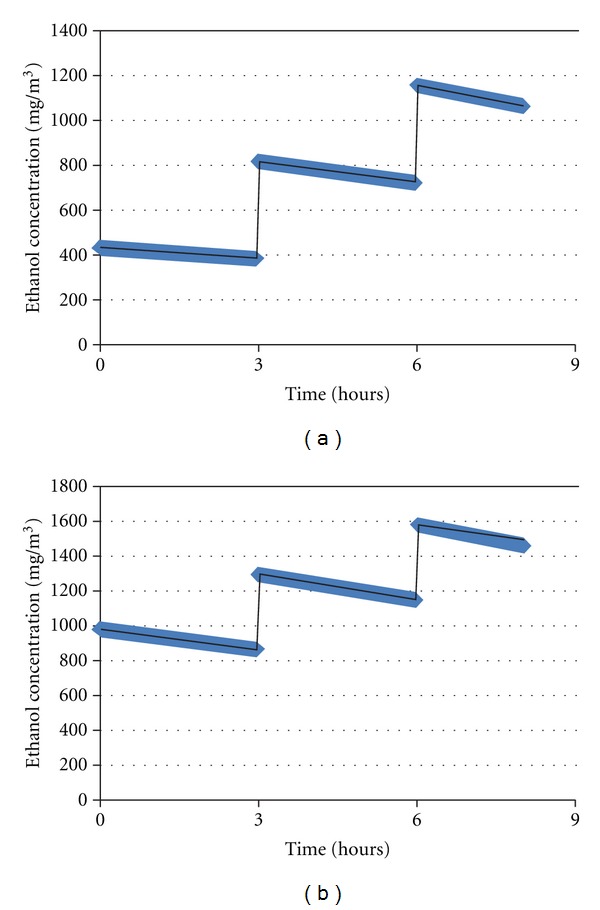
(a) Atmospheric ethanol concentration (day 1). By using the AIHA software, the ethanol atmospheric emission was calculated as follows: 30 rubbing hands (RH) at time 0 h, 30 RH at 3 h and 30 RH at 6 h resulting in a mean ethanol atmospheric concentration of 408 mg/m^3^ (time 0–3 h), 768 mg/m^3^ (time 3–6 h), and 1108 mg/m^3^ (time 6–8 h). (b) Atmospheric ethanol concentration (day 2). By using the AIHA software, the ethanol atmospheric emission was calculated as follows: 30 rubbing hands (RH) at time 0 h, 30 RH at 3 h, and 30 RH at 6 h resulting in a mean ethanol atmospheric concentration of 924 mg/m^3^ (time 0–3 h), 1224 mg/m^3^ (time 3–6 h), and 1518 mg/m^3^ (time 6–8 h).

The air ethanol concentration was estimated using the AIHA software [4]. As mechanical ventilation was defective, several maximizing scenarios were used considering a low air change rate of 0.08 m^3^/min (Figures 2(a) and 2(b)). In addition, it was also considered that for each simultaneous 30 frictions, an atmospheric ethanol emission of 50.4 g of ethanol was calculated, resulting in an atmospheric concentration of 0.43 g/m^3^.

Changes over time for worst case scenario make it possible to predict the following peaks of exposure.

#### 3.1.1. During the First Day

At time 0 (*T*_0_), the atmospheric concentration after the first frictions was 0.43 g/m^3^.

At *T*_3h_, it was 0.81 g/m^3^, coming from both the second frictions (0.43 g/m^3^) and the residual concentration of 0.38 g/m^3^ from the first frictions at *T*_0_.

At *T*_6h_, the atmospheric concentration was 1.15 g/m^3^ corresponding to the sum of the atmospheric concentration resulting from the frictions at *T*_6h_ (0.43 g/m^3^) and the residual concentration of 0.72 g/m^3^ present in the atmosphere after the preceding frictions.

#### 3.1.2. During the Second Day

At *T*_0h_, the atmospheric concentration was 0.97 g/m^3^ (sum of the frictions at *T*_0h_ (0.43 g/m^3^) and the remaining residual concentration from all the night of 0.54 g/m^3^). At *T*_3h_, the atmospheric concentration was 1.23 g/m^3^ (sum of frictions at *T*_3h_ (0.43 g/m^3^) and the residual concentration of 0.8 g/m^3^). At *T*_6h_, the atmospheric concentration was 1.58 g/m^3^ (sum of 0.43 g/m^3^ and 1.15 g/m^3^, as residual atmospheric concentration).

In conclusion, these results made possible to predict the atmospheric ethanol mean concentration after two successive days: 408 mg/m^3^ (time 0–3 h), 768 mg/m^3^ (time 3–6 h), 1108 mg/m^3^ (time 6–8 h) for the first day and 924 mg/m^3^ (time 0–3 h), 1224 mg/m^3^ (time 3–6 h), and 1518 mg/m^3^ (time 6–8 h) for the second day (Figures 2(a) and 2(b)). It was considered a total of 378 mL of ethanol (i.e., 180 frictions of 3 mL or 540 mL EBHS at 70% ethanol) in a classroom volume of 116 m^3^, under the worst case scenario of a defective ventilation giving a low air change rate of 0.08 m^3^/min.

### 3.2. Blood Ethanol Concentration Calculation

Blood ethanol concentration induced by these exposures is predicted using a physiologically based toxicokinetic model to simulate inhalation route exposure to various airborne concentrations of ethanol and to predict its toxicokinetic behaviour [5].

PBPK modeling of ethanol takes into account physicochemical and biochemical parameters to predict blood ethanol over time following exposure. This model was developed by Schlouch and Tardif [6].

In our study, the maximum blood ethanol concentration was estimated to reach a plateau at 5.9 mg/L when breathing air with an ethanol mean concentration of 924 mg/m^3^ (time 0–3 h), 1224 mg/m^3^ (time 3–6 h), and 1518 mg/m^3^ (time 6–8 h) (the maximal occupational exposure concentration) (Table 2). 

## 4. Discussion

The absorbed ethanol is found in the blood in variable proportions, depending on the route of exposure. Several epidemiological studies show that alcoholic beverages consumption increases the cancer risk in human [8]. Harmful effects on reproduction and development in the liver and in the central and peripheral nervous system have also been observed. These effects can be observed after ingestion of 12 g of ethanol per day (i.e., the equivalent of one glass of wine) and leading to a peak blood from 150 to 250 mg/L, which represents relatively high levels of blood ethanol concentration (ethanolaemia peak). In our study, the low elevation of blood ethanol concentration (5.9 mg/L) obtained by simulation in the patient, which remains within the limit values of endogenous blood ethanol concentration in nondrinker humans [9], does not seem to be a trigger of AP, on the basis of the current knowledge.

Several AP's cases reported in the literature are in relation with consumption of alcoholic beverages by oral route. High blood concentrations are more likely after drinking alcoholic beverages. The elimination rate is dependent on whether person is an alcoholic with an induced metabolism or not. The metabolite acetaldehyde is very reactive and may be responsible for some of the harmful effects of ethanol.

The AP causes are multiple and remain undetermined in 15 to 25% of cases [10]. In western countries, migration of gall stones in the biliary tract is the main cause (38%). In our study, the negativity of exploration by radiological scan does not confirm the role of gall stones. Alcohol abuse is the second leading cause (36%). The results of a meta-analysis published in 2009 [11] highlighted an exponential dose-response relationship between average volume of alcohol consumption and pancreatitis. Overall, the results indicate a nonlinear association between alcohol consumption and the relative risk of pancreatitis [11]. The risk curve between alcohol consumption and pancreatitis was relatively flat at low levels of alcohol consumption, and it markedly increased with increasing levels of consumption. It is nonexistent among low alcohol consumers (up to 2 drinks per day or 24 g of alcohol), occurring in individuals consuming 36 g alcohol per day and only becoming statistically significant for a daily consumption of 48 g of alcohol. Alcohol causes an AP in 10% of very excessive drinkers (more than 80 g alcohol per day). The drugs come in third place and are involved in about 5% of AP. There is no semiological criterion specific of a drug AP even if hydrochlorothiazide can cause acute pancreatitis, as it has been reported in the literature for this drug. In addition, many other causes have been described, of low frequency and some still discussed [12]: genetic predisposition, congenital malformation of the pancreas, tumours, infection, hypertriglyceridemia, hypercalcemia, pregnancy, and so on.

In the current state of knowledge, the side effects of ethanol, related to chronic exposure by dermal absorption or inhalation route, have not sufficiently been documented in humans. A retrospective study in connection with the dermal use of alcohol-based hand sanitizers conducted by the French Poison Control Centers in 2009 reported that side effects listed are mostly related to misuse [13]. In addition, apart from this case, no other AP has been reported in link with the use of ethanol as EBHS.

The likelihood that dermal exposure increases plasmatic concentration was excluded right away for the reason that our earlier risk assessment and other several studies showed a very low to negligible dermal absorption, even after intensive use of EBHS. It is generally allowed that, on nondamaged skin, about 1% of the dose of ethanol initially placed on the surface actually penetrates the skin barrier. The amount is thus negligible when compared to pulmonary absorption, which is estimated at 60% [14, 15].

Indeed, in the literature, several studies have been conducted to explore the dermal absorption issue [16–19] and concluded that ethanol skin absorption does not increase blood ethanol concentration significantly. In the Afssaps's risk assessment opinion published in 2009 [1], all these data relative to the exposure by dermal route was analyzed and the conclusion retained put forward that the low or negligible absorption allows to conclude for an absence of the risk for the consumer even after an excessive use.

Inhalation of ethanol vapors at normal atmospheric concentrations will thus not result in any significant blood concentration [20]. The metabolic elimination of ethanol from the blood will in most cases exceed the uptake.

Lester and Greenberg [21] showed that inhalation of ethanol vapor does not seem to cause any severe acute effects at ethanol concentrations below 10,000 mg/m^3^. However, headache and cough have been reported after about 30 minutes of inhaling ethanol vapor at concentrations of 2600 and 3400 mg/m^3^, respectively. When the concentration increases, the airways become irritated with resulting cough, lachrymation, and breathing difficulties [22].

In case ethanol vapor exposure, the manifestation of undesirable effects require that the ethanol reaches in one hand plasma and in the other hand target organ. The concentration in the plasma depends on the concentration in air, the exposure duration, the breathing rate, the absorption of ethanol across the lung, the basal metabolism, and also the elimination rate of ethanol. 

In this study, the situation of confinement and exposure to ethanol by inhalation route showed that maximum blood ethanol amounted to 5.9 mg/L. The results are consistent with studies in the literature. Indeed, Campbell and Wilson [23] found after exposure to ethanol vapor concentration of 1900 mg/m³ in the air for 3 h a slight elevation of blood ethanol by repeated measurements at 0, 35, 60, 120, and 180 minutes limited to less than 2 mg/L. Miller et al. [19] conducted a study on five volunteers working at the hospital emergency department rubbing their hands 50 times in four hours using a EBHS containing 62% ethanol. Blood ethanol remains below 0.5 mg/L. In addition, an estimation of blood ethanol by modeling in the Afsset's risk assessment report [20] showed that, after 42 frictions with a EBHS containing 80% ethanol, repeated 8 hours, it was estimated at 1.28 mg/L, within the range of endogenous blood ethanol concentration. Pendlington et al. [24] conducted three studies whose aim was to determine the rate of evaporation and absorption of ethanol and to estimate the dermal penetration of ethanol *in vitro* on pig skin and *in vivo* in humans in 16 volunteers using an aerosol spray. The results of these studies indicate a short half-life of evaporation (about 11 seconds) and skin absorption strongly increased by the occlusion. The *in vitro* study does not determine a dermal absorption rate. Thus, the results in humans have shown no detectable blood ethanol at the limit of detection of 9 mg/L and this after evaporation of a quantity of up to 17.28 g of an aerosol constituted of 44% ethanol.

In other hand, malaise and headache recorded of 9 persons at day six after the two successive-day demonstration remained unclear, one of the possible explanations is probably due to the symptoms collective type of discomfort related to the syndrome of chemical smell intolerance [25]. This concerns subjects exposed to low concentrations of aerial contaminants, with various symptoms suggesting the entanglement of objective reasons not well understood with psychological subjective factors. Based on low exposure (atmospheric and/or systemic) concentration to ethanol predicted, it is not possible to relate rationally the effect observed with EBHS uses.

Using a physiologically based toxicokinetic model to simulate inhalation route in this study, the systemic concentration of 5.9 mg/mL predicted is not easily related to the acute pancreatitis reported. A study conducted in 1557 nondrinkers volunteers showed that the endogenous blood ethanol is related to the synthesis of ethanol by microorganisms in the digestive tract which is between 0 and 35.2 mg/L, with a mean of 1.1 mg/L and median of 0.4 mg/L. The endogenous ethanol seems produced by yeast fermentation and other intestinal microorganisms and/or restored by food. Indeed, for example, certain fruit juices can contain up to 3 g ethanol/L [26] and an apple juice 2 g/L. Recall that a glass of alcohol (10–12 g of ethanol) leads to a peak blood from 150 to 250 mg/L and drunkenness can appear only from 1.5 g/L of ethanol [27, 28].

Finally, in general family and personal history, clinical symptoms, biochemical analysis, and/or radiological tests as scan identify the majority of aetiologies of AP. In our study, the Afssaps suggested further investigations in the absence of track on the origin of the AP. Thus, the etiological investigation is worth pursuing in order to properly rule out a tumour origin.

## 5. Conclusion

Our study shows the ethanol exposure under the conditions of defective mechanical ventilation in the classroom resulted in a maximum blood ethanol concentration of 5.9 mg/L determined by PBPK modeling, which is considered in the same order of magnitude as the endogenous serum ethanol. According to Al-Awadhi et al. [9], the endogenous ethanol level could be reaching an average of 1.1 mg/L (0 to 35 mg/L) after absorption of ethanol synthesized by digestive tract microorganisms. As example, each glass of alcoholic beverage contains about 12 g of ethanol [28] and leads after oral absorption to a pick plasma ethanol concentration at about 250 mg/L.

Under the conditions of this study no relationship can be made between the exposure to EBHS and the increase of blood ethanol concentration being able to lead to the occurrence of the reported acute pancreatitis.

## Figures and Tables

**Table 1:** Model parameters for the ethanol PBPK.

Physiological parameters of the model PBPK for ethanol	

Body weight	70
Cardiac output (L/h/kg)^0.75^	18
Alveolar ventilation (L/h/kg)^0.75^	18
Absorbed fraction	0.62

Fraction of cardiac output to each compartment	

Fat	0.05
Liver	0.25
Rapidly perfused	0.39
Slowly perfused	0.19
Brain	0.12

Total	1.00

Fraction of body volume compartments	

Fat	0.213
Liver	0.0257
Rapidly perfused	0.0443
Slowly perfused	0.607
Brain	0.02

Total	0.91

Physicochemical and metabolic parameters	

*Partition coefficients*	
Blood: air	2280
Fat: air	226
Liver: air	1730
Rapidly perfused: air	2030
Slowly perfused: air	1710
Brain: air	1870

Liver metabolic parameter	

Metabolism rate (mg/h/kg)^0.75^	359.5
Affinity constant (mg/L)	82.1

**Table 2:** Blood ethanol concentration predictions (BECPs) based on 8 h exposure average (mg/L). The BECP was calculated by using the PBPK model of ethanol with the following exposure condition previously estimated. *For the day 1*, the atmospheric ethanol concentration was 408 mg/m^3^ (time 0–3 h), 768 mg/m^3^ (time 3–6 h), and 1108 mg/m^3^ (time 6–8 h). *For the day 2*, the atmospheric ethanol concentration was 924 mg/m^3^ (time 0–3 h), 1224 mg/m^3^ (time 3–6 h), and 1518 mg/m^3^ (time 6–8 h).

		*T* (h)	BECP
Blood ethanol concentration (mg/L)	Day 1	0	0.00
		8	4.21
	Day 2	24	0.00
		32	5.9
